# Mechanism of covalent binding of ibrutinib to Bruton's tyrosine kinase revealed by QM/MM calculations†

**DOI:** 10.1039/d0sc06122k

**Published:** 2021-01-28

**Authors:** Angus T. Voice, Gary Tresadern, Rebecca M. Twidale, Herman van Vlijmen, Adrian J. Mulholland

**Affiliations:** Centre for Computational Chemistry, School of Chemistry, University of Bristol Cantock's Close Bristol BS8 1TS UK adrian.mulholland@bristol.ac.uk; Computational Chemistry, Janssen Research & Development, Janssen Pharmaceutica N. V. Turnhoutseweg 30 B-2340 Beerse Belgium

## Abstract

Ibrutinib is the first covalent inhibitor of Bruton's tyrosine kinase (BTK) to be used in the treatment of B-cell cancers. Understanding the mechanism of covalent inhibition will aid in the design of safer and more selective covalent inhibitors that target BTK. The mechanism of covalent inhibition in BTK has been uncertain because there is no appropriate residue nearby that can act as a base to deprotonate the cysteine thiol prior to covalent bond formation. We investigate several mechanisms of covalent modification of C481 in BTK by ibrutinib using combined quantum mechanics/molecular mechanics (QM/MM) molecular dynamics reaction simulations. The lowest energy pathway involves direct proton transfer from C481 to the acrylamide warhead in ibrutinib, followed by covalent bond formation to form an enol intermediate. There is a subsequent rate-limiting keto–enol tautomerisation step (Δ*G*^‡^ = 10.5 kcal mol^−1^) to reach the inactivated BTK/ibrutinib complex. Our results represent the first mechanistic study of BTK inactivation by ibrutinib to consider multiple mechanistic pathways. These findings should aid in the design of covalent drugs that target BTK and other similar targets.

## Abstract 2

QM/MM simulations show that covalent modification of BTK by ibrutinib proceeds *via* an intramolecular proton transfer from C481 to the acrylamide warhead of ibrutinib, followed by covalent bond formation and subsequent keto–enol tautomerisation.

## Graphical abstract

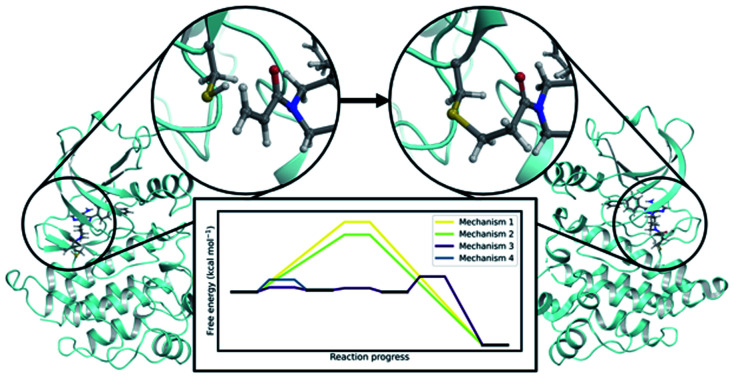

## Introduction

Covalent inhibitor drug discovery has re-emerged because of advantages compared with conventional non-covalent reversible binding that can include complete target blockage, increased selectivity and longer duration of action.^1–3^ Recent years have seen the approval of several new marketed covalent drugs targeting protein kinases.^4,5^ In particular, inhibition of Bruton's tyrosine kinase (BTK) is an attractive target for blood cancers and autoimmune diseases, due to its function in signal transduction in the B-cell antigen receptor (BCR) pathway.^6,7^ BTK inhibitors have also been explored as possible inhibitors against the SARS-CoV-2 coronavirus in drug repurposing studies.^8^ Ibrutinib and acalabrutinib are two BTK inhibitors that are approved for the treatment of B-cell cancers including mantle cell lymphoma (MCL) and chronic lymphocytic leukaemia (CLL).^9^ Both drugs contain electrophilic Michael acceptor warheads that covalently modify a cysteine residue (C481) in the kinase domain of BTK. Utilising warheads of this type to target poorly conserved cysteine residues is a common technique to developing covalent inhibitors in drug discovery.^10^ Despite the massive investments made to discover and develop these drugs, the detailed mechanism of covalent binding to BTK is unknown. Understanding the precise mechanism will help in the design of improved covalent inhibitors targeting BTK, and also other covalent drug targets. The ability to rationally tune covalent reactivity should lead to safer, more selective covalent drugs that have fewer side effects.^11,12^

The reaction of a Michael acceptor warhead such as an acrylamide group to a thiol side chain is typically modelled in three steps. First, deprotonation of the cysteine thiol occurs to form a thiolate anion, followed by nucleophilic attack of the thiolate on to the electrophile to form an enolate intermediate, and finally re-protonation of the enolate to form a covalent thiol-adduct.^13^ Sulfur reactivity in proteins has been studied previously using combined quantum mechanics/molecular mechanics (QM/MM) approaches.^1,14^ Effort has focused on cysteine proteases and protein kinases, given their roles in some disease processes. For example, covalent nitrile inhibitors of the cysteine protease rhodesain have been investigated using QM/MM reaction simulations at the semi-empirical PM6 level.^15^ The protocol was found to be a useful predictor of reversible covalent binding affinity, in good agreement with experimental data. The mechanism of covalent modification of C797 by an acrylamide warhead in the protein kinase EGFR has been elucidated by QM/MM modelling at the self-consistent-charge density-functional-based tight-binding (SCC-DFTB) level.^16,17^ These results identified a neighbouring aspartate residue, D800 in the *i*+3 position relative to C797, as the catalytic base to deprotonate the cysteine thiol.

**Fig. 1:**
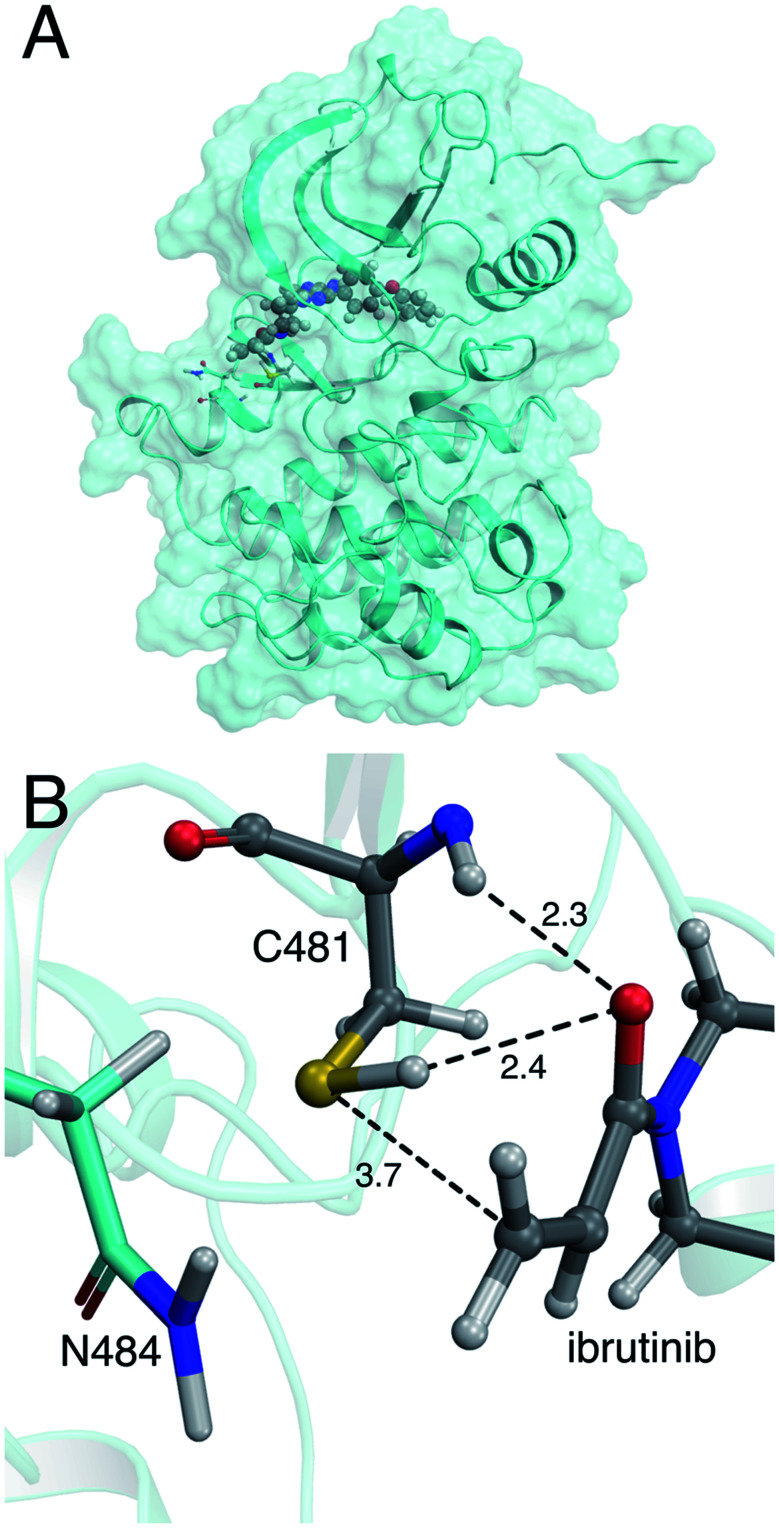
(A) Crystal structure of the BTK kinase domain with ibrutinib bound in the ATP binding pocket (PDB 5P9I). (B) Binding mode of ibrutinib in the active site of BTK, in a reactive conformation observed in MM molecular dynamics simulations. The acrylamide warhead is positioned in close proximity to C481. The S–C distance is 3.7 Å. The asparagine residue found in the *i*+3 position, N484, is also shown. Important active site distances are reported in Å.

Comparing kinases that contain cysteine at the equivalent position to C481 in BTK, different amino acids are found at the *i*+3 position: the majority are either aspartate (Asp) or asparagine (Asn).^18^ BTK contains an Asn residue, N484, rather than an Asp residue or other good proton acceptor in the *i*+3 position (Fig. 1). Asparagine is a very weak base, suggesting a mechanism in BTK different to the EGFR Asp-catalysed mechanism. Residues in the surrounding microenvironment can modulate cysteine p*K*_a_, depending on their properties.^19^ Although there is no experimentally determined C481 p*K*_a_ available, the p*K*_a_ of a free cysteine thiol in solution is 8.6. The cysteine p*K*_a_ calculated for EGFR in a recent computational study is 11.1.^20^ In comparison, with the *i*+3 residue in BTK being Asn rather than Asp, a slightly more acidic cysteine p*K*_a_ of 10.4 was predicted in the same study. A p*K*_a_ of around 10 would result in a 1 : 1000 ratio of ionised to neutral cysteine at pH 7, with a free energy difference of 4.1 kcal mol^−1^ between the ionised and neutral states. Thus, C481 is predominantly neutral at physiological pH. This suggests that a nearby proton acceptor may be required for deprotonation of the thiol group, either prior to or concerted with the reaction with the electrophile. Although asparagine performs catalytic roles in some enzymes, for example as a nucleophile in some protein splicing reactions,^21,22^ the amide sidechain of Asn is weakly basic and has only been reported to act as a base when it is activated by metal ions.^23^ It is therefore unlikely to accept a proton from C481 in BTK indicating that a different mechanism of inhibition operates in BTK than that which has been established for EGFR.

**Fig. 2:**
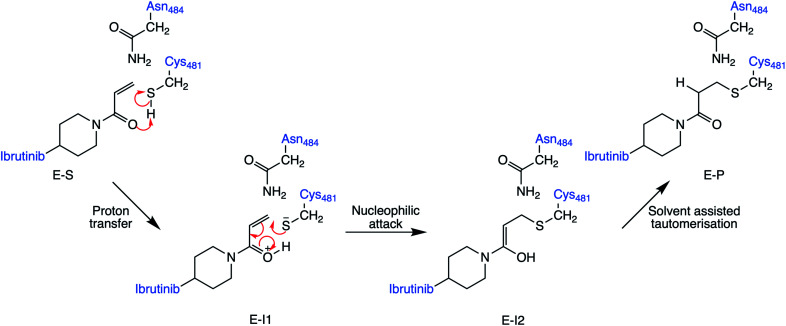
Lowest energy pathway of BTK inhibition by covalent inhibitor ibrutinib from QM/MM umbrella sampling simulations at the DFTB3/MM level. Other mechanisms were also explored (see ESI†) but found to have significantly higher barriers to reaction.

The lack of an obvious amino acid to act as acid or base leaves several other possible mechanisms for covalent inhibition of C481 in BTK by Michael acceptors. These include mechanisms that proceed *via* direct addition between C481 and the Michael acceptor (Fig. 2), such as a direct transfer of the thiol proton to the α-carbon of the acrylamide warhead to produce the covalently bound keto adduct in a single step. Alternatively, the thiol proton could transfer to the carbonyl oxygen atom of the acrylamide inhibitor to form an enol intermediate, followed by a tautomerization step to form the covalently bound keto product. There is also the possibility that water could be involved in the reaction, *e.g.* assisting in proton transfer between the cysteine thiol and acrylamide warhead. Here, we assess each of these pathways using a QM/MM umbrella sampling approach. The results identify the probable mechanism of covalent binding between the acrylamide warhead in ibrutinib and BTK.

## Methods

QM/MM umbrella sampling molecular dynamics (MD) simulations were used to explore each mechanistic pathway in the kinase domain of BTK. The QM region included all of the ibrutinib ligand and the side chain of C481. The effects of including N484 in the QM region were also tested; this did not significantly change the calculated energetics, showing the choice of QM region to be reasonable^24^ (Fig. S8, ESI†). For the water-mediated pathways, an adjacent crystallographic water molecule hydrogen bonded to the carbonyl oxygen of the acrylamide was also included in the QM region. The umbrella sampling protocol consisted of generating a free energy surface (FES) using the density-functional tight-binding (DFTB3) QM method, with 20 ps of MD sampling per reaction coordinate (RC) window to get an approximate minimum energy pathway (MEP) for the reaction. This was followed by running an additional 10 ps of sampling along the minimum energy path at the same level of theory. Previous studies have compared the use of DFTB, PM3 and PM6 methods for modelling the reactivity of thiol compounds.^13,25^ Our extensive benchmarking (ESI, Fig. S1–S7†) showed that PM3/6/7 performed poorly for modelling thiol addition mechanisms, whereas DFTB3 gave geometries in good agreement with higher level ab intio (MP2) and DFT methods. In all cases, tests showed that the FESs were converged with respect to simulation time, with good overlap between neighbouring simulation windows (ESI, Fig. S8 and S9†).

## Results and discussion

QM/MM umbrella sampling MD simulations indicate that the lowest energy pathway of C481 modification by ibrutinib proceeds by a direct proton transfer (PT) from the C481 thiol group to the carbonyl oxygen atom of ibrutinib, resulting in a Cys-S^−^/C=OH^+^ ion pair (E-I1, Fig. 2). This is followed by C–S bond formation to form a covalent enol complex (E-I2, Fig. 2). Finally, a solvent-assisted keto–enol tautomerization step forms the covalent keto product (E-P, Fig. 2). This pathway is lower in energy than the three alternative pathways we investigated (see ESI for details†), including solvent-assisted PT resulting in enol formation; a direct 1,2-olefin addition pathway; and a solvent-assisted variant of the 1,2-olefin addition pathway. The mechanism that we identify is consistent with experimental kinetics (see below).

The free energy surface for the lowest energy pathway has low free energy barriers of 3.1 kcal mol^−1^, 2.6 kcal mol^−1^, and 10.5 kcal mol^−1^ for the initial PT step, S–C bond formation and solvent-assisted tautomerization steps, respectively. Invoking a water molecule to assist in the initial proton transfer step is entropically unfavorable, resulting in a higher barrier than a direct PT (8.4 kcal mol^−1^*vs.* 3.1 kcal mol^−1^). An equivalent solvent-assisted PT and enol formation has been modelled for the modification of cysteine residues by microcystins, where a high barrier of 21.9 kcal mol^−1^ was calculated for the reaction pathway involving water.^26^ The 1,2-addition pathway, consisting of a direct PT from the thiol to the α-carbon of the acrylamide warhead and simultaneous S–C formation has been reported as a high energy pathway by Rowley *et al.* who calculated the barrier of the 1,2-σ addition of methylvinyl ketone and methyl thiolate to be 65.2 kcal mol^−1^ at the CCSD(T)//ωB97X-D level.^27^ QM/MM simulations of 1,2-addition in BTK suggested a very high barrier (approximately 47.7 kcal mol^−1^), and we therefore discounted it as a feasible mechanism based on our simulations and the findings of Rowley *et al.* We also investigated the solvent-assisted variant of this pathway, but the free energy surface did not represent a feasible reaction pathway: we observed additional PTs that indicated a strong preference for the reaction to proceed *via* an enol intermediate (see ESI for details†).

**Fig. 3:**
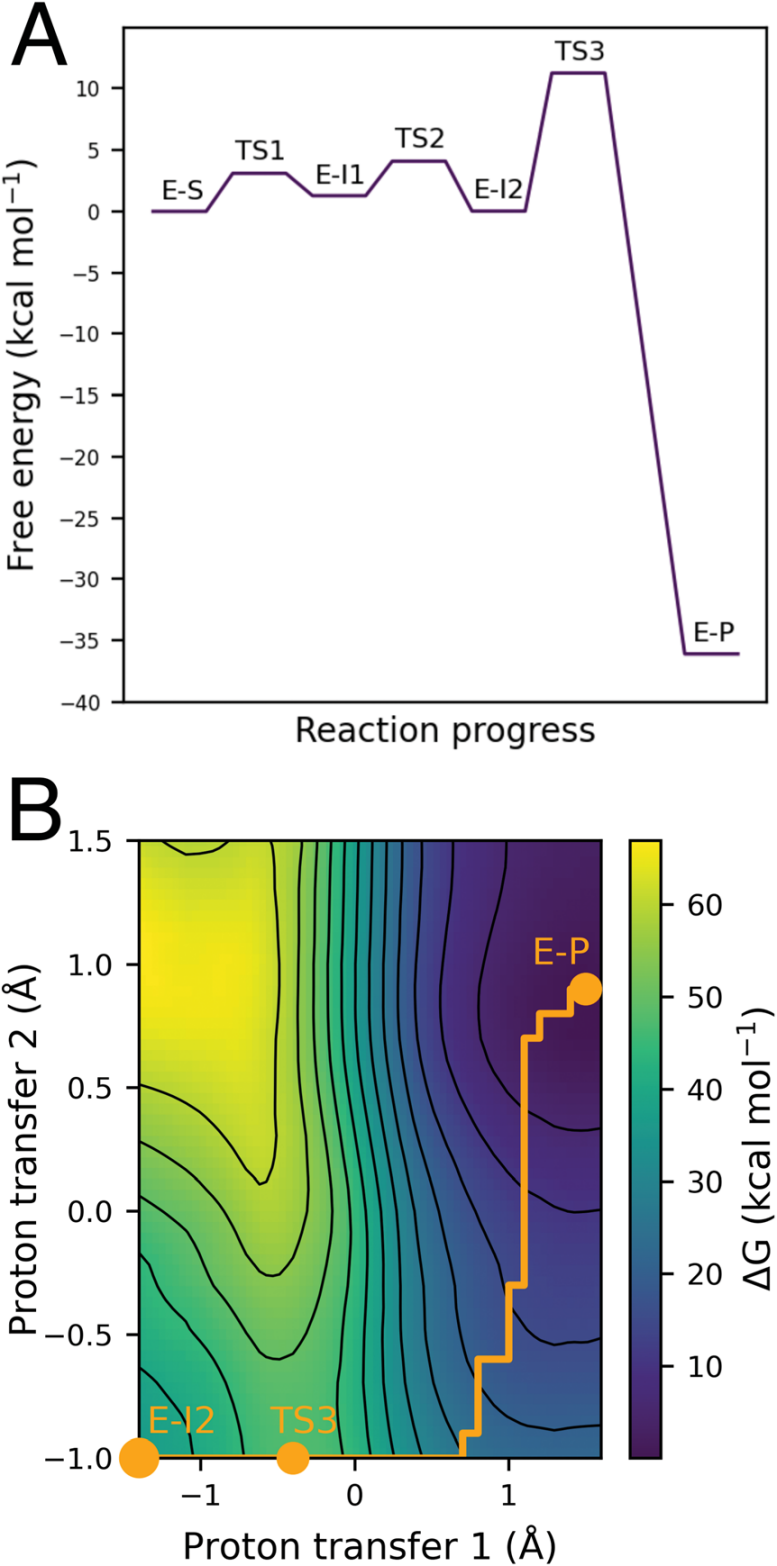
(A) Free energy profile for reaction of BTK with ibrutinib calculated at the DFTB3/MM level. The lowest energy pathway consists of a direct proton transfer step, followed by S–C bond formation. (B) The final step is a rate-limiting solvent-assisted keto–enol tautomerization with a free energy barrier of 10.5 kcal mol^−1^ for this step.

**Fig. 4:**
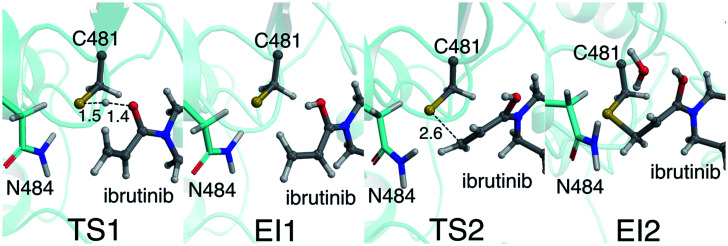
Representative structures of TS1, EI1, TS2 and EI2 from QM/MM umbrella sampling MD at the DFTB3/MM level from the lowest energy pathway. Transition state distances are reported in Å. The stabilising interaction between the C481 thiolate and the N484 side chain is clearly visible in EI1 and TS1.

Our QM/MM simulations also show the importance of the *i*+3 asparagine residue. Although the N484 sidechain does not directly participate as a proton acceptor in the reaction (unlike the *i*+3 aspartate residue in EGFR),^16,17^ it has an essential role in stabilising the TS and intermediates along the reaction pathway in BTK (Fig. 4). This is particularly evident in the initial proton transfer step, in which this asparagine side chain interacts strongly with the developing negative charge on the C481 sulfur atom as the proton transfer occurs. Without the N484/C481-S^−^ interaction, no stable intermediate was found for E-I1 (see ESI†). To investigate the possibility that the protonated acrylamide group (E-I1, Fig. 2) might be favoured by the umbrella sampling restraints, 25 ps of unrestrained QM/MM MD was run on the E-I1 structure at the DFTB3/MM level. Over the course of the trajectory, no proton transfers occurred, and the structure did not collapse, indicating that it is indeed a stable intermediate. The stabilizing N484/C481-S^−^ interaction continues until TS2 is reached, at which point the N484/C481-S^−^ interaction breaks, as the S–C bond begins to form between the thiol group and the inhibitor (Fig. 3). The stabilization of the thiolate by N484 and water molecules, in combination with a more favorable TS geometry for the stepwise pathway, helps to explain why the barriers for the PT step and S–C formation steps are low (Fig. 4). In EGFR, a low barrier of 8.6 kcal mol^−1^ has been calculated for S–C bond formation between C797 and an acrylamide inhibitor at the DFTB3/MM level.^16^ In that EGFR study, desolvation of the thiolate anion prior to nucleophilic attack was found to be an important reactivity determinant. In BTK, the negatively charged thiolate is stabilized by only two water molecules, compared to three in EGFR, possibly contributing to the lower reaction barrier in BTK.

There are ten kinases that contain a cysteine residue in the equivalent position to BTK.^28^ Of these, TEC kinase contains an *i*+3 asparagine residue analogous to BTK. Ibrutinib exhibits very similar inactivation kinetics with TEC and BTK.^29^ Comparison of the experimental kinetics of ibrutinib with kinases that contain an *i*+3 aspartate residue, such as EGFR and ITK, shows a very different kinetic profile. This indicates an alternative mechanism of action in kinases with Asn at the *i*+3 position could occur. Covalent binding of ibrutinib to kinases that contain *i*+3 aspartate residues apparently proceed *via* a base-catalysed mechanism, where proton transfer occurs *via* the aspartate residue as previously shown *e.g.* by a similar detailed QM/MM studies for EGFR.^16,17^ The base-catalysed mechanism that operates in EGFR is expected to have a higher *k*_inact_.^17^ However, IC_50_ measurements show that ibrutinib is a better inhibitor of BTK than of EGFR.^30^ Overall inhibition will be determined by the *k*_inact_/*K*_i_ ratio, which is two orders of magnitude higher for BTK than EGFR.^30^ This implies that the inhibition of BTK depends on a high non-covalent binding affinity, which should also be considered in inhibitor design.^17,31^ Although S–C formation is the rate-limiting step in kinases with an *i*+3 aspartate residue, its replacement by asparagine in BTK results in a different mechanism in which S–C bond formation is no longer the rate limiting step. The *i*+3 residue is crucial to dictate the mechanism of reaction. Site-directed mutagenesis of the *i*+3 residue may provide a tool to test this hypothesis.

Keto–enol tautomerization steps have been previously reported to be high energy pathways and thus unlikely to occur in thio-Michael addition reactions in protein active sites.^32^ However, our QM/MM simulations show that this is a feasible reaction step in BTK covalent inhibition due to the low free energy barrier and the solvent-exposed nature of the edge of the ATP binding pocket. Both of the crystal structures available for BTK complexed with ibrutinib (PDB codes: 5P9I and 5P9J^33^) contain a water molecule positioned above the carbonyl oxygen atom (*d*[O_ibrutinib_–O_wat_] = 2.9 Å) and the α-carbon (*d*[O_wat_–Cα_ibrutinib_] = 3.6 Å) ideally placed for a solvent-assisted tautomerization. QM/MM umbrella sampling MD simulations also indicate that the enol intermediate E-I2 forms a hydrogen bond with a water molecule (Fig. 4). The FES at the DFTB3/MM level for this step (Fig. 3) shows a reaction barrier of 10.5 kcal mol^−1^. This is in good agreement with a previous study that investigated the energetics of solvent assisted keto–enol tautomerization in a substituted triazolone compound, and found a reaction barrier of 10.6 kcal mol^−1^ at the B3LYP level.^34^ The covalent keto adduct lies 36.8 kcal mol^−1^ lower in energy than the enol intermediate, consistent with irreversible inhibition.

Experimental studies of BTK inhibition kinetics are available that provide inactivation rates (*k*_inact_) of several covalent BTK inhibitors.^35^ These are summarized in Table 1, along with the corresponding Δ*G*^‡^ values, calculated using the Eyring equation. The similarity of *k*_inact_ for inhibitors with different chemical scaffolds and reactive warheads suggests little dependence on the ligand structure. For ibrutinib, the inactivation rate corresponds to a Δ*G*^‡^ value of 19.6 kcal mol^−1^. This is higher than the calculated barrier heights from our QM/MM simulations, due to two main factors. First, comparisons with higher level calculations shows that DFTB3 QM method underestimates the barrier to reaction. For S–C bond formation, DFTB3 gives barriers ∼5 kcal mol^−1^ lower than those predicted by higher level (ω-B97-XD/6-31G(d)) QM/MM umbrella sampling MD simulations (Fig. S14, ESI†). Second, there is likely to be a free energy cost associated with forming a reactive conformation.^36,37^ The Δ*G*^‡^ value derived from experimental kinetics will include any free energy penalty for adopting a reactive conformation. This potentially includes rotation of the amide side chain of N484, and also rotation of the thiol side chain of C481 to form a reactive conformation. Taking these two factors into account, the calculated energetics are consistent with experimental kinetics.

**Table 1:** Inactivation rates of 5 covalent BTK inhibitors.^35^ The corresponding free energy of activation Δ*G*^‡^ values (calculated using transition state theory) are shown for comparison

Inhibitor	BTK inactivation rate, *k*_inact_ (s^−1^)	Free energy of inactivation Δ*G*^‡^ (kcal mol^−1^)
Ibrutinib	2.66 × 10^−2^	19.6
Acalabrutinib	5.59 × 10^−3^	20.5
Zanubrutinib	3.33 × 10^−2^	19.5
Spebrutinib	1.36 × 10^−2^	20.0
Tirabrutinib	9.72 × 10^−2^	20.2

Our tests of QM methods (see ESI†) show that DFTB3 underestimates the reaction barrier for C–S bond formation compared to higher levels of QM theory such as ω-B97-XD and MP2. However, DFTB3 predicts reaction pathways that are structurally in close agreement with higher level methods (*e.g.* in intrinsic reaction coordinate calculations (Fig. S4†)). DFTB3 provides a reasonably accurate description of the energetics of the rate-limiting solvent-assisted keto–enol tautomerization step (Fig. S7†). The balance of speed and accuracy afforded by DFTB3 therefore make it an appropriate method for the assessment of possible reaction pathways, while using a relatively large QM region (including the drug molecule). During the final preparation of this work, a preprint appeared that includes modelling of the mechanism of covalent binding of a cyanoacrylamide inhibitor to BTK.^31^ That work studied a mechanism involving direct attack of the (deprotonated) Cys thiolate on the ligand electrophile. The barrier to S–C bond formation was predicted to be 3.4 kcal mol^−1^, and is consistent with our barrier for this step in the reaction.

## Conclusions

Our results indicate that the most probable mechanism for BTK inhibition by ibrutinib involves three steps. An initial proton transfer occurs from the thiol group to the carbonyl oxygen atom of the acrylamide group of ibrutinib (Δ*G*^‡^ = 3.1 kcal mol^−1^). This is followed by S–C bond formation to form an enol intermediate (Δ*G*^‡^ = 2.6 kcal mol^−1^). A rate-determining solvent-assisted tautomerization step then occurs to form the covalently bound BTK/ibrutinib complex (Δ*G*^‡^ = 10.5 kcal mol^−1^). This pathway was lower in energy than all the other pathways that were investigated (see ESI† for full details of consideration of alternative mechanisms).

Understanding the precise mechanism by which C481 in BTK is covalently modified by ibrutinib should help in the design of safer, more selective, covalent drugs. To our knowledge, there are currently no other studies that have investigated the covalent mechanism of action of ibrutinib at the atomic level. Insights from this work should help to rationally tune the reactivity of acrylamide (and potentially other types of) covalent inhibitors of BTK. Our simulations highlight the importance of inhibitor conformation, thiol reactivity, and the hydration of the binding site. Common medicinal chemistry strategies could be employed to enhance or attenuate covalent reactivity. These include the use of substituted acrylamides with different electronic properties, alternative linker groups to attach the electrophilic warhead to the main drug scaffold, or using different covalent reactive groups/warheads.^38^ However, the kinetic data for five covalent BTK inhibitors shown in Table 1 suggest that even changes in the linker and/or different warheads have virtually no effect on the observed inactivation rates. This raises the possibility that reactivity is influenced by the protein itself, *e.g.* affecting the orientation and p*K*_a_ of the cysteine residue. Designing new inhibitors that modulate cysteine p*K*_a_*in situ*, or affect it is conformational behaviour, could therefore be useful for tuning covalent reactivity and designing specific inhibitors.

## Conflicts of interest

There are no conflicts to declare.

## Supplementary Material

SC-012-D0SC06122K-s001
