# Supplementary material for: Mechanism of covalent binding of ibrutinib to Bruton's tyrosine kinase revealed by QM/MM calculations
Source: Chem Sci. 2021 Jan 28;12(15):5511–6. doi: 10.1039/d0sc06122k (PMC8097726; doi:10.1039/d0sc06122k)
Supplement: SC-012-D0SC06122K-s001 [file SC-012-D0SC06122K-s001.pdf]

Electronic Supplementary Material (ESI) for Chemical Science.
This journal is © The Royal Society of Chemistry 2021

Supporting Information

Mechanism of covalent binding of ibrutinib to Bruton's tyrosine kinase revealed by QM/MM calculations

Angus T. Voice [a], Gary Tresadern [b], Rebecca M. Twidale [a], Herman van Vlijmen [b], Adrian J. Mulholland [a]

[a] Centre of Computational Chemistry, School of Chemistry, Cantock's Close, University of Bristol, Bristol BS8 1TS, United Kingdom. [b] Computational Chemistry, Janssen Research & Development, Janssen Pharmaceutica N. V., Turnhoutseweg 30, B-2340 Beerse, Belgium

Table of Contents

S1.2 Model setup 7

S1.3 QM/MM protocol 8

S1.4 Umbrella sampling convergence 10

S1.5 Alternative mechanistic pathways 12

S1.5.1 Mechanism 1 12

S5.1.2 Mechanism 2 13

S5.1.3 Mechanism 3 14

S5.1.4 Mechanism 4 15

S5.1.5 Summary 16

References 17

S1.1 Method validation

We performed 1D potential energy scans of a model system comprising methyl thiolate and an acrylamide warhead attached to a piperidine linker as an ibrutinib mimic using Gaussian16.[1] Geometry optimisations were performed along the covalent C-S bond formation reaction coordinate, that was varied between 1.6 and 3.0 Å in steps of 0.01 Å. The results suggested that B3LYP does not predict a stable minimum for the carbanion intermediate (Figure S1). This is in agreement with previous studies that show significant failings for B3LYP and that Range-Separated DFT Functionals are Necessary to Model Thio-Michael Additions.[2,3] In addition, DFTB2 and DFTB3//DFTB2 incorrectly predict a carbanion intermediate at S-C distances of 2.45 Å (Figure S2). DFTB2 geometries are used because there is no DFTB3 implementation in Gaussian16. The energy jump observed in the potential energy surface scans for AM1, PM3 and PM6 at large S-C distances is the result of isomerisation of the olefin group in acrylamide.

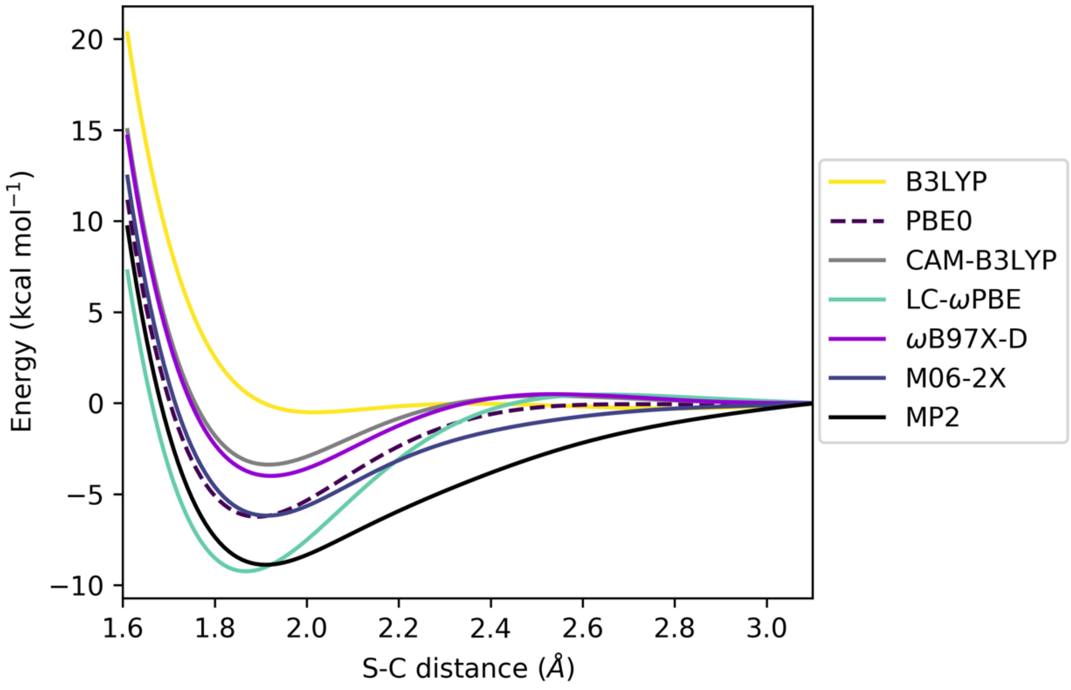

Figure S1. Potential energy profiles of the nucleophilic attack step in thiol-Michael addition between methyl thiolate and acrylamide at the DFT/aug-cc-pVTZ level. All methods tested show a stable enolate intermediate, apart from B3LYP/aug-cc-pVTZ in accordance with previous studies.[2,3]

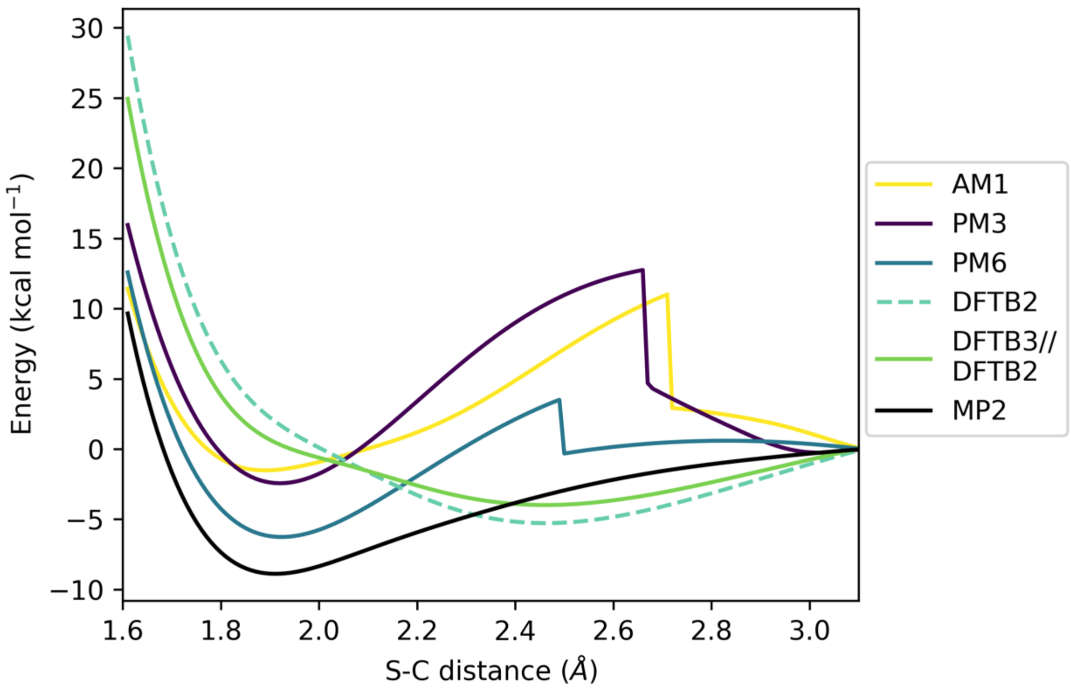

Figure S2. Potential energy profiles of the nucleophilic attack step in thiol-Michael addition between methyl thiolate and acrylamide computed using semi-empirical quantum chemistry methods. The potential energy profile at the MP2 level of theory is shown for comparison. The energy jump observed in the potential energy surface scans for AM1, PM3 and PM6 at large S-C distances is the result of isomerisation of the olefin group in acrylamide.

To test the accuracy of the geometries predicted for C-S bond formation predicted by PM6 and DFTB3//DFTB2 along the reaction path, we performed single point energy calculations on the structures along the DFTB3//DFTB2 and PM6 reaction pathways with the ωB97X-D density functional in combination with the aug-cc-pVTZ basis set (denoted ωB97X-D//DFTB2 and ωB97X-D//PM6), Figure S3. The potential energy scan for S-C formation at the ωB97X-D//DFTB2 level is very similar to the potential energy scan at the ωB97X-D/aug-cc-pVTZ level, indicating that the geometries predicted by DFTB2 are reliable and are suitable for modelling thiol addition reactions. This was not the case at the ωB97X-D//PM6 level, where the PM6 geometries led to approximately 10-15 kcal mol^{-1} higher energies across the surface.

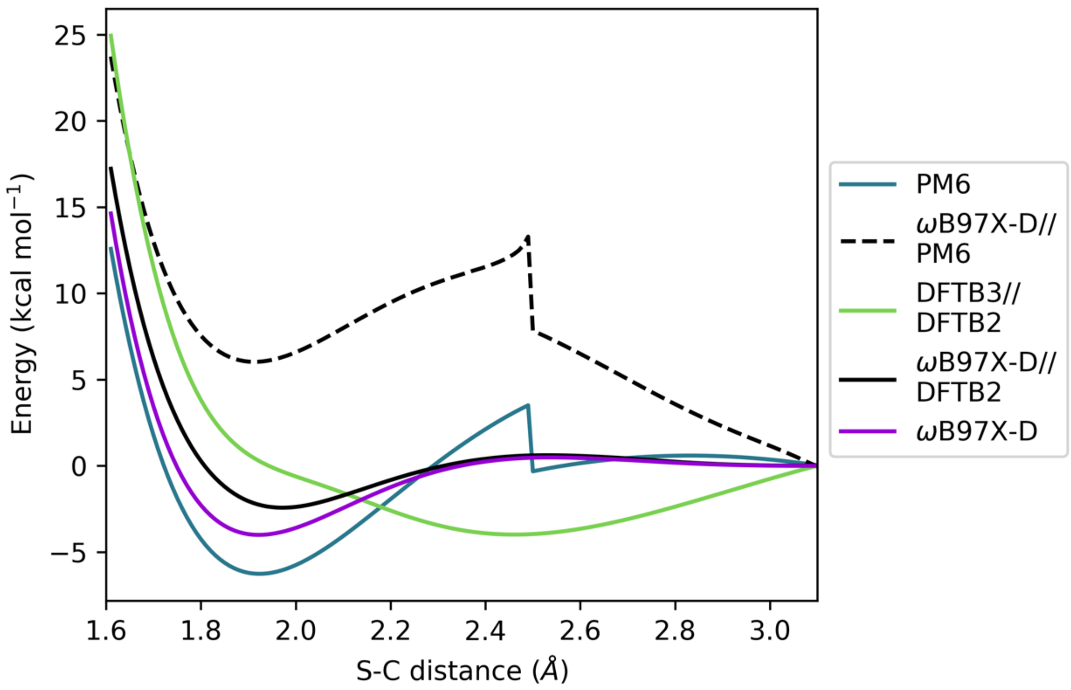

Figure S3. Potential energy profiles of the nucleophilic attack step in the thiol-Michael addition between methyl thiolate and acrylamide computed with ωB97X-D single point calculations on DFTB2 and PM6 geometries. Potential energy profiles optimised at the ωB97X-D, PM6 and DFTB3//DFTB2 levels of theory are shown for comparison.

Next, we carried out a transition state (TS) search for the reaction shown in Figure S6(B) with a variety of density functional and semi-empirical QM methods. We confirmed the presence of a TS by checking for a single imaginary frequency corresponding to the reaction coordinate and performed intrinsic reaction coordinate (IRC) calculations to investigate the pathways predicted by different methods for thiol addition by plotting the value of the reaction coordinate for S-C bond formation and proton transfer from methyl thiol to the carbonyl oxygen of the truncated ibrutinib model (Figure S4). All of the density functional methods and DFTB2 predict a similar reaction pathway along the nucleophilic attack and proton transfer reaction coordinates. AM1 was in reasonably close agreement, whereas PM3, PM6 and PM7 were in poor agreement with the density functional methods and predicted significantly different reaction pathways in which the C-S bond formation occurs prior to proton transfer. The corresponding energy plots for each IRC pathway (Figure S5) suggest that DFTB2 underestimates the barrier to S-C formation and proton transfer. These results confirm the suitability of using the DFTB3 method for modelling thiol addition reactivity of this type.

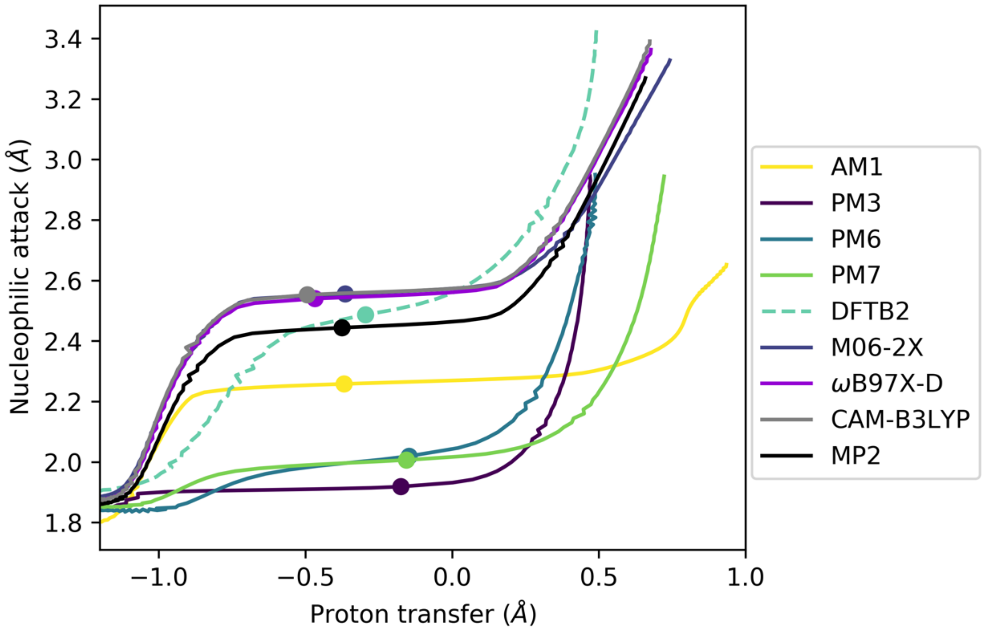

Figure S4. Plot to show the nucleophilic attack and proton transfer reaction coordinates along the reaction path predicted by IRC calculations with several different QM methods for mechanism **3** (the lowest energy pathway), where the thiol proton transfers to the carbonyl oxygen of acrylamide to form an enol. The location of transition states for each method are shown as circles on each pathway.

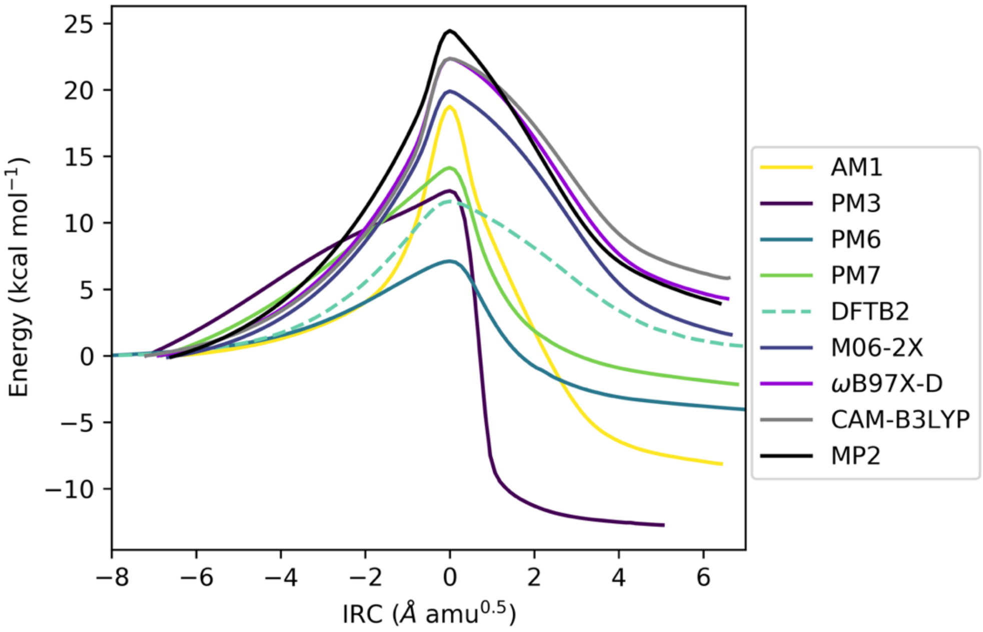

Figure S5. Energy plot of the IRC calculations suggest that DFTB2 underestimates the barrier to S-C formation and proton transfer in a model BTK/ibrutinib complex (Figure S6(B)).

The IRC pathway at the DFTB3 level (Figure S4) was in better agreement with higher level methods than that for S-C bond formation alone (Figure S2). Examination of the structures and important bond lengths of the TSs for DFTB3, MP2 and ωB97X-D (Figure S6 and Table S1) shows a high degree of structural similarity between the TSs. The S-C and O-H distances predicted by DFTB3 are closer to the values predicted by MP2 than the values predicted by ωB97X-D (Table S1). In addition, the IRC for solvent-assisted tautomerisation confirms that DFTB3 gives energetics of this reaction step similar to ωB97X-D, showing that DFTB3 provides a good description of the reaction (Figure S7).

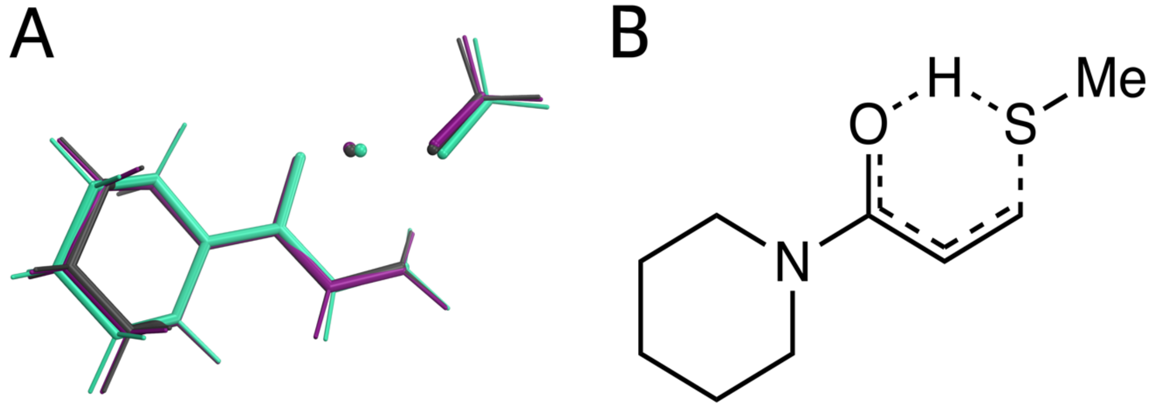

Figure S6. (A) Optimised gas phase transition state structures of the structure shown in (B) at the DFTB2 level (green), ωB97X-D/6-31G(d) level (purple) and MP2/6-31G(d) level (black). The 6-31G(d) basis set was chosen to match the basis set used in the umbrella sampling protocol.

Table S1. Tabulated bond lengths in the optimised TS corresponding to the model BTK/ibrutinib system (Figure S6(B)).

Distance (Å)	DFTB3	ωB97X-D	MP2
**S-C**	2.47	2.53	2.44
**O-H**	1.20	1.15	1.20
**S-H**	1.59	1.72	1.68

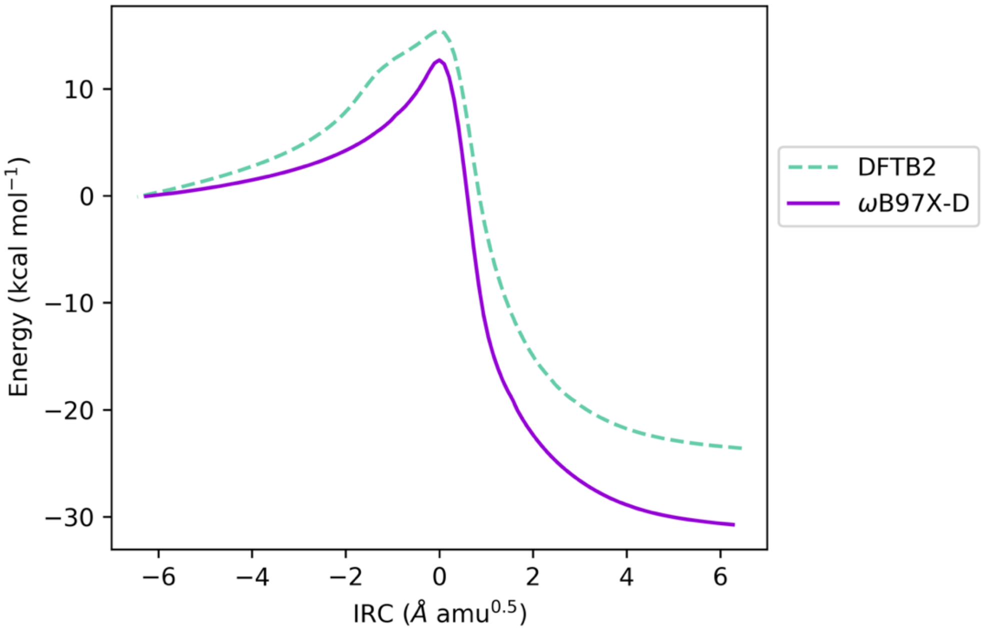

Figure S7. Potential energy profiles from IRC calculation of the rate-limiting solvent-assisted tautomerisation step on a model BTK/ibrutinib system (Figure S6(B)) in the gas phase at the DFTB2 and level and ωB97X-D/6-31G(d) level. The DFTB2 energies are in good agreement with ωB97X-D/6-31G(d) for this solvent-assisted tautomerisation reaction.

S1.2 Model setup

Snapshots for the QM/MM reaction simulations were selected from 500 ns of classical molecular dynamics simulations using the AMBER ff14SB force field. Structural coordinates of the covalently bound BTK/ibrutinib complex were taken from the crystal structure with PDB ID 5P9J.[4] Missing residues were included by using the Modeller program.[5] Hydrogens atoms were added using the protein preparation wizard in Maestro,[6] where PROPKA3.1[7,8] was used to assign protonation states of titratable amino acid side chains and optimise the hydrogen bonding network. For the covalently bound C481-ibrutinib complex, RESP charges were generated using the REDServer[9] (RESP ESP charge Derive Server) and any missing FF parameters were generated using Antechamber, distributed the AMBER 2018 package.[10] The SOLVATE program[11] developed by H. Grubmüller and V. Groll was used to solvate the system by creating a solvation shell of 5 Å of TIP3P water around the protein using 8 Gaussians. The Amber program tleap was then used to create a truncated octahedron with a padding of 5 Å and a closeness of 0.75. Na^+ and Cl^- ions were added to create a salt concentration of 0.1 M.

The system was then subject to minimisation, then heating from 0 to 293.15 K over 75 ps with a weak 5 kcal mol^{-1} restraint on backbone CA atoms using Langevin dynamics and a collision frequency of 5 ps^{-1}. Equilibration was then performed in the NPT ensemble, using Langevin dynamics and a Monte Carlo barostat to maintain the pressure at 1.01325 bar with a pressure relaxation time of 1 ps. The weak backbone restraints were gradually released during this equilibration phase, before running 500 ns of production MD in the NPT ensemble using the same pressure and regulation settings as the equilibration phase.

S1.3 QM/MM protocol

QM/MM reaction simulations were performed by starting from a representative snapshot of the covalently bound enzyme-inhibitor complex selected from MM MD simulations. Each mechanistic pathway was explored in the backwards direction (towards the non-covalently bound enzyme-substrate complex).[12] Four possible reaction mechanisms for the covalent modification of C481 by ibrutinib were investigated:

- **Mechanism 1**: Direct keto product formation
- **Mechanism 2**: Solvent-assisted keto product formation
- **Mechanism 3**: Direct enol formation, and subsequent keto-enol tautomerisation to form the final covalently bound keto product
- **Mechanism 4**: Solvent-assisted enol formation, and subsequent keto-enol tautomerisation to form the final covalently bound keto product

The free energy surfaces were generated using the weighted histogram analysis method (WHAM), using code distributed by the Grossfield lab.[13,14]

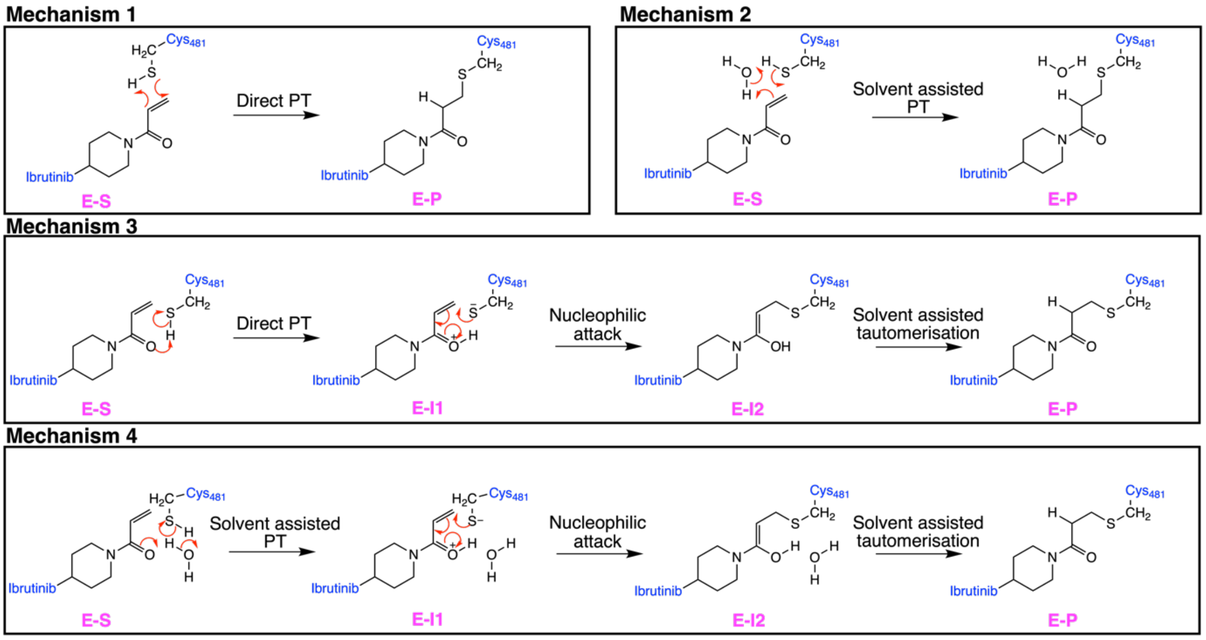

Scheme S1. Schematic of the four mechanistic pathways explored for the covalent inhibition of C481 in BTK by ibrutinib.

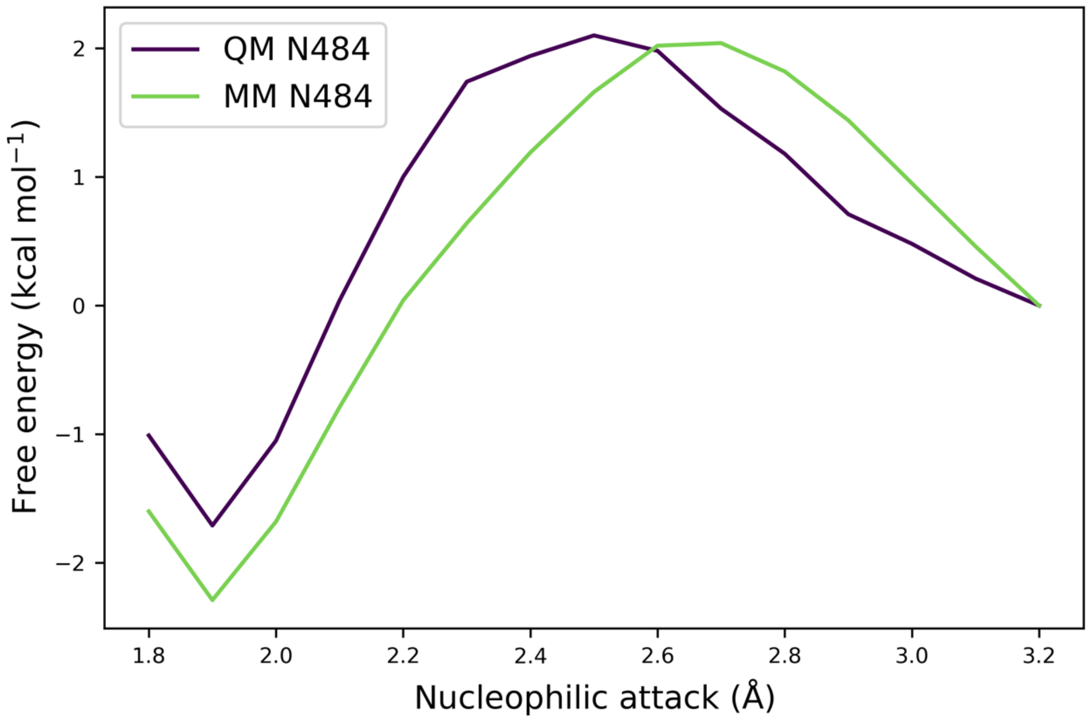

Figure S8. QM/MM free energy profile of S-C formation between C481 in BTK and ibrutinib when N484 is included in the QM region and the MM region when 25 ps of sampling are performed in each umbrella sampling window. The difference in barrier height is 0.1 kcal mol^{-1}.

S1.4 Umbrella sampling convergence

To check convergence of the umbrella sampling pathways, histograms of the number of counts of each sampled reaction coordinate value in each reaction coordinate window were produced.[15,16] An example of the histograms along the proton transfer and S-C reaction coordinates from mechanism **3** (the lowest energy pathway) are shown below. These show good overlap between neighbouring umbrella sampling windows and uniform heights of each histogram.

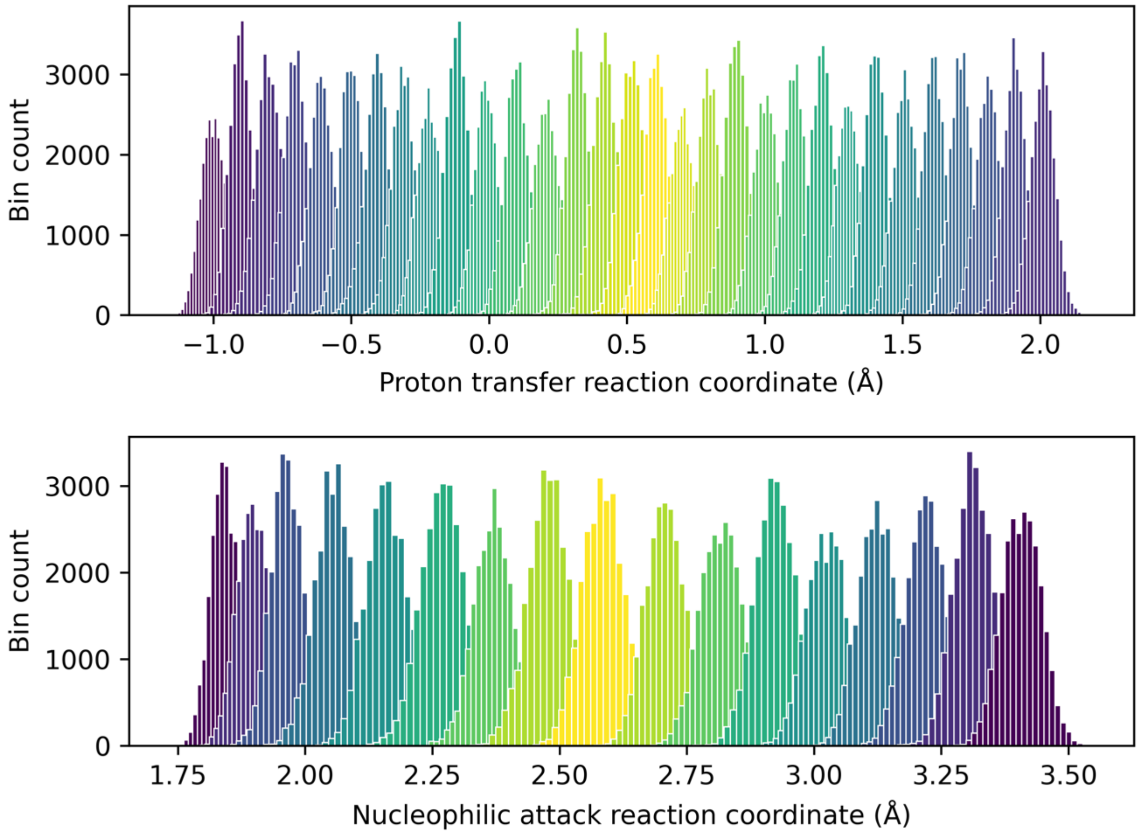

Figure S9. Umbrella sampling histograms along the proton transfer and nucleophilic attack (S-C formation) reaction coordinates for mechanism **3** after 25 ps of sampling in combination with a biasing potential of 200 kcal mol^{-1} Å^{2} in each umbrella sampling window.

We also checked that the barrier heights had converged by performing WHAM analysis on the complete dataset and 80% of the dataset). The free energies were considered converged if the change in the barrier height ($\Delta G^{\ddagger}$) was less than 0.1 kcal mol^{-1}. The free energy change in each reaction coordinate window when comparing 100% and 80% of the sampling data across the entire free energy surface for the solvent-assisted tautomerisation step is less than

0.4 kcal mol^{-1}, and the free energy change in the barrier height is 0.02 kcal mol^{-1} (Figure S10). These tests show that the QM/MM umbrella sampling results are well converged.

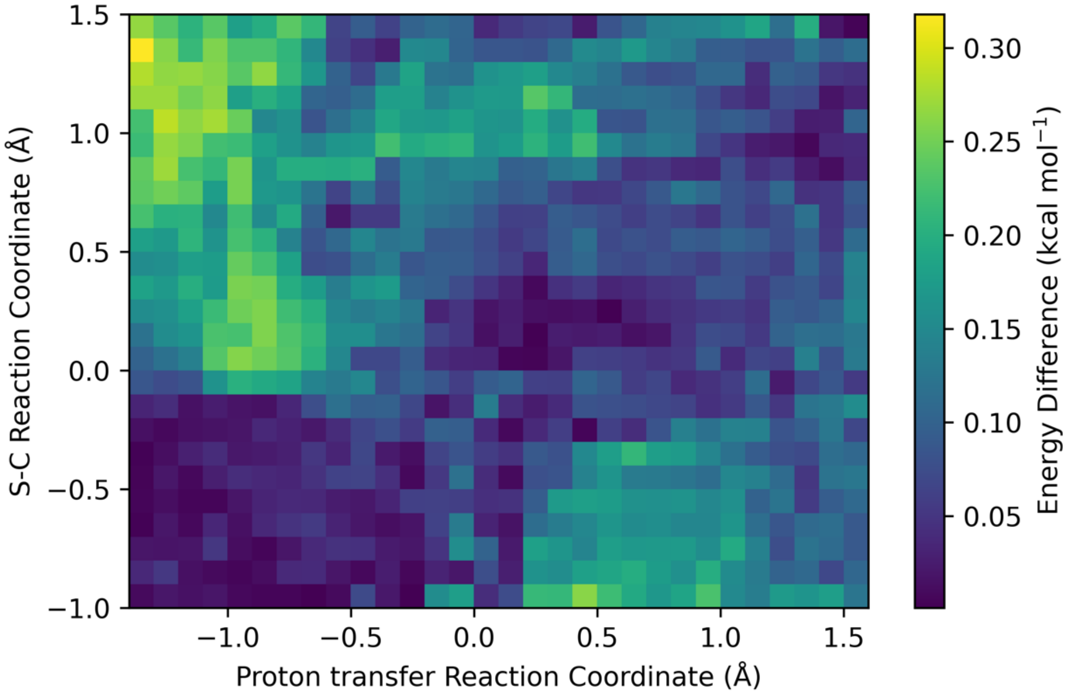

Figure S10. Heat map to show the difference in free energy in each reaction coordinate window for the solvent-assisted tautomerisation free energy surface (mechanisms **3** and **4**) when 100% and 80% of sampling data are compared at the DFTB3/MM level of theory.

S1.5 Alternative mechanistic pathways

S1.5.1 Mechanism 1

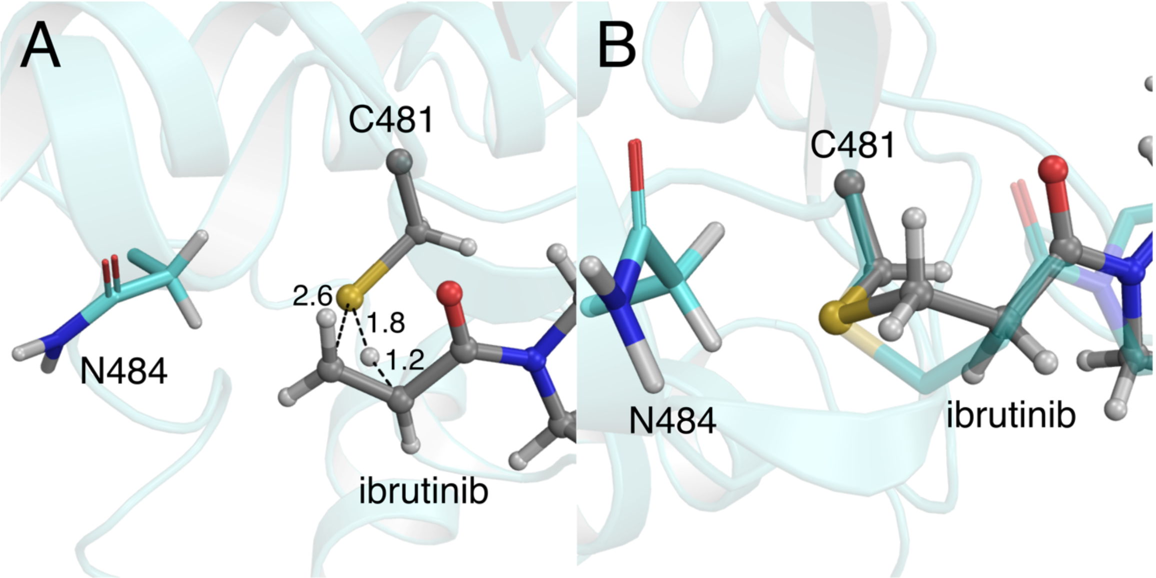

Figure S11. Approximate transition state, taken from the final frame of the highest energy reaction coordinate window (RC1 = 2.6 Å, RC2 = 0.6 Å) along the reaction pathway of Mechanism 1. (B) Representative snapshot of the covalently bound BTK/ibrutinib keto product (EP) from mechanism **1**. The geometry around the newly formed S-C bond differs from the crystal geometry (PDB 5P9J,[4] shown in transparent green), and results in a high energy product state.

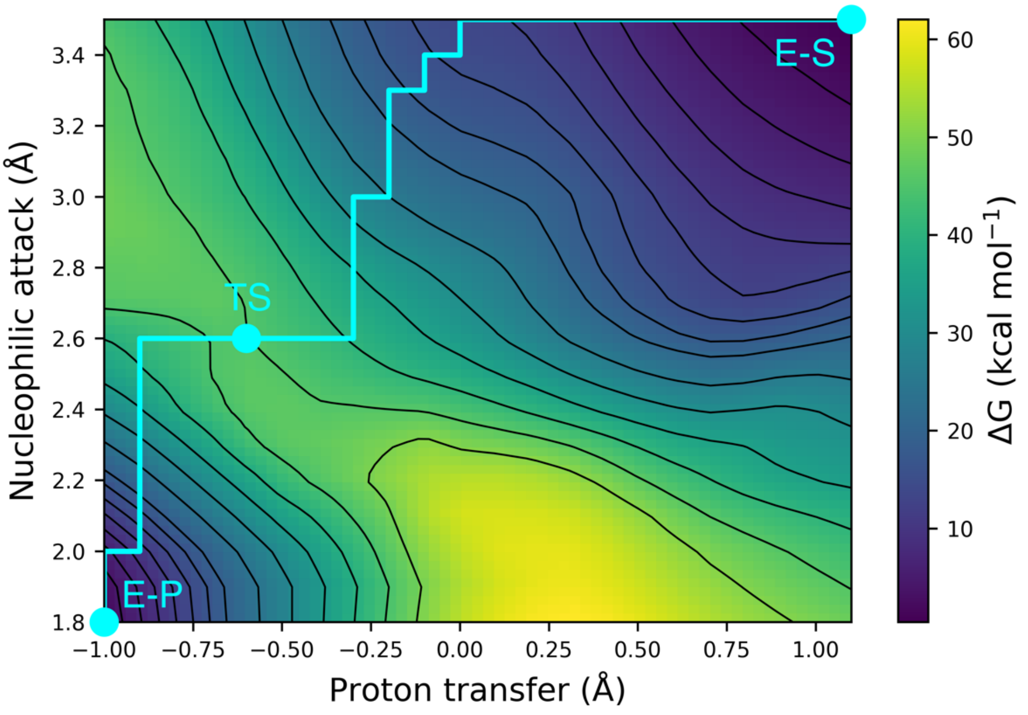

Figure S12. Free energy surface produced at the DFTB3 level, with 30 ps of sampling per window along the minimum energy path for the direct addition of ibrutinib to C481 in BTK. The high reaction barrier of 47.7 kcal mol^{-1} is consistent with the reaction being thermally forbidden.

S5.1.2 Mechanism 2

For mechanism **2**, we had trouble generating a productive reaction pathway and were only able to successfully generate an approximate 2D free energy pathway along the diagonal between each reaction coordinate. Reaction coordinate (RC) 1 was defined by proton transfer from the α-carbon of the inhibitor to the oxygen of a water molecule and S-C bond formation. RC2 was defined by an additional proton transfer from the same water molecule to the C481 sulfur atom. Exploration of the remainder of the free energy surface resulted in additional proton transfers, even when additional restraints were added. As a result, the free energy barrier for mechanism **2** is approximate, but the numerous failed attempts to obtain a productive pathway, and the high reaction barrier suggest this is not a feasible reaction mechanism for covalent modification of C481 by ibrutinib.

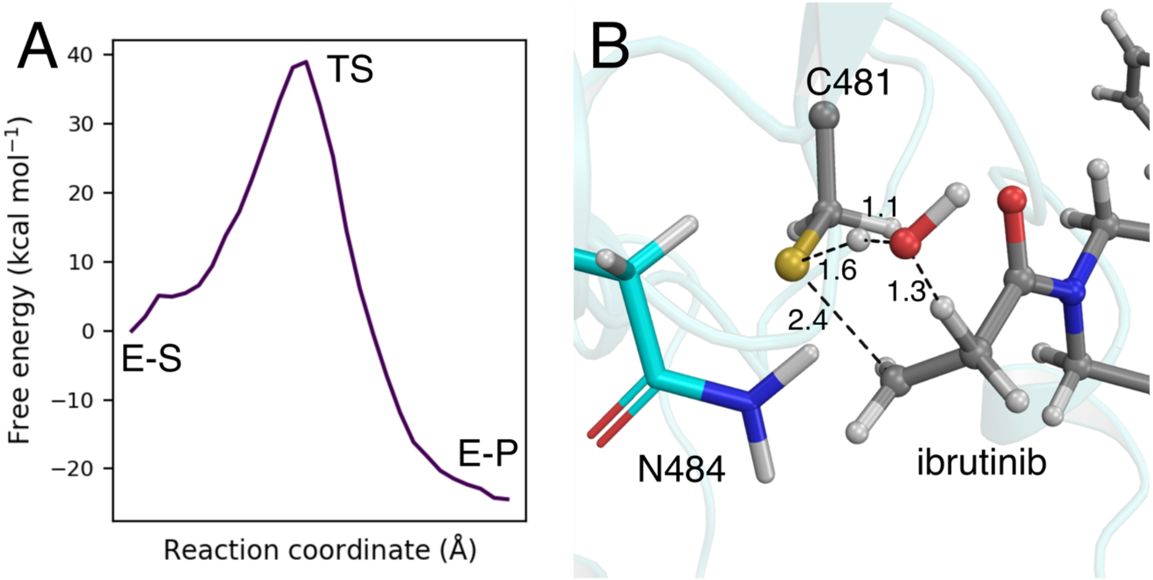

Figure S13. (A) Approximate free energy profile for mechanism **2**, where solvent assisted thiol addition occurs to result in the keto product. The approximate free energy barrier is 39.0 kcal mol^{-1} and 2 ps of sampling was carried out in reach umbrella sampling window. (B) Approximate transition state for mechanism **2**. The structure is the final frame from the highest energy reaction coordinate window along the reaction path.

S5.1.3 Mechanism 3

In order to confirm that the formation of the C481-S^{-}/N484-NH_2 interaction is dependent on the orientation of the amide side chain of N484, rather than the QM method used, free energy surfaces were produced at the DFTB3 and ωB97X-D/6-31G(d) levels of theory (Figure S14). The starting points for both simulations were a single snapshot in which N484 was oriented away from C481. The reaction was modelled in the forwards direction (from ES to EI2). Comparison of the free energy surfaces produced at the DFTB3 and ωB97X-D/6-31G(d) levels of theory reveals that no stable EI1 intermediate is predicted by either method as a result of the absence of a C481-S^{-}/N484-NH_2 interaction. The barrier to enol formation is 23.9 and 29.2 kcal mol^{-1} for DFTB and ωB97X-D/6-31G(d) respectively, indicating that DFTB underestimates the barrier for S-C formation by approximately 5 kcal mol^{-1}. The reaction barrier is much higher when the reaction is modelled as a concerted process due to unfavorable geometry of the TS, and the absence of the stabilising interaction from N484.

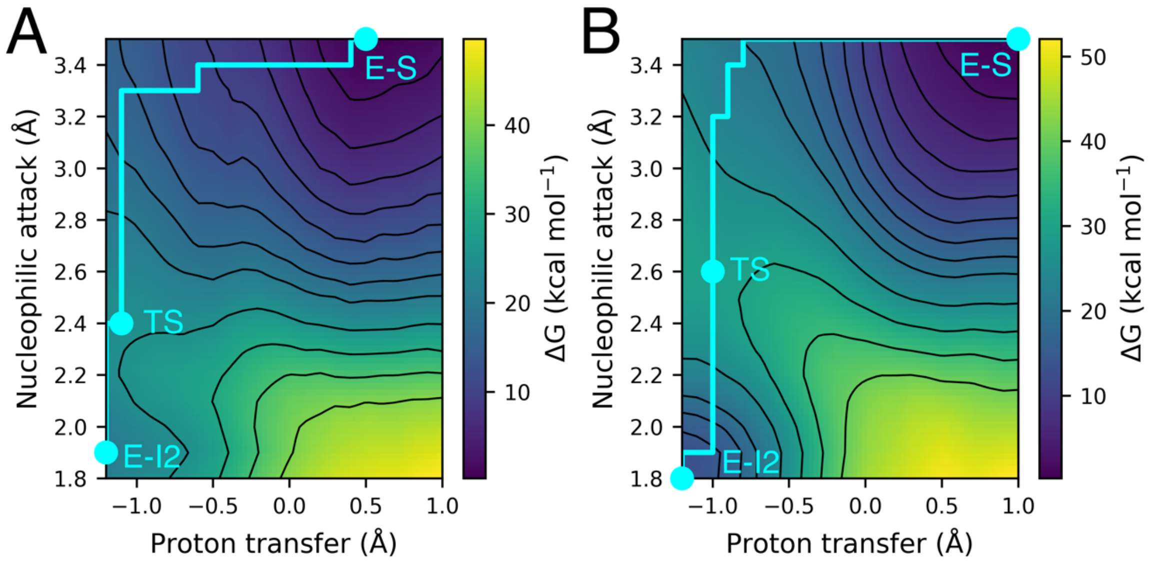

Figure S14. Free energy surfaces for the mechanism **3** reaction between C481 and the acrylamide warhead of covalent drug ibrutinib when no N484 interaction is present at the DFTB3/MM level (A) and ωB97X-D/6-31G(d)/MM level (B). Due to the computational cost of performing MD at the DFT level, only 2 ps of sampling in each reaction coordinate window were performed. The barrier heights for the reaction are 23.9 and 29.2 kcal mol^{-1} for the DFTB and ωB97X-D surfaces respectively. DFTB3 underestimates the reaction barrier by approximately 5 kcal mol^{-1} compared to ωB97X-D.

S5.1.4 Mechanism 4

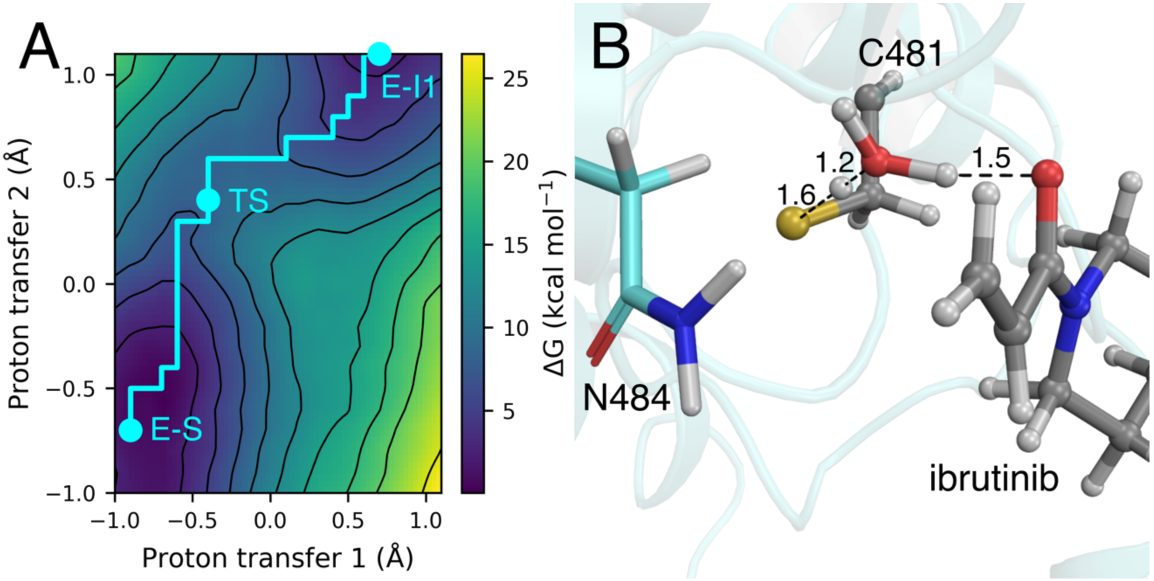

Figure S15. Free energy surface for the solvent assisted proton transfer from C481 in BTK to the carbonyl oxygen atom of the covalent inhibitor ibrutinib at the DFTB3/MM level. In each reaction coordinate window 30 ps of sampling was performed along the minimum energy path, and the barrier to the proton transfer was 8.3 kcal mol^{-1}.

S5.1.5 Summary

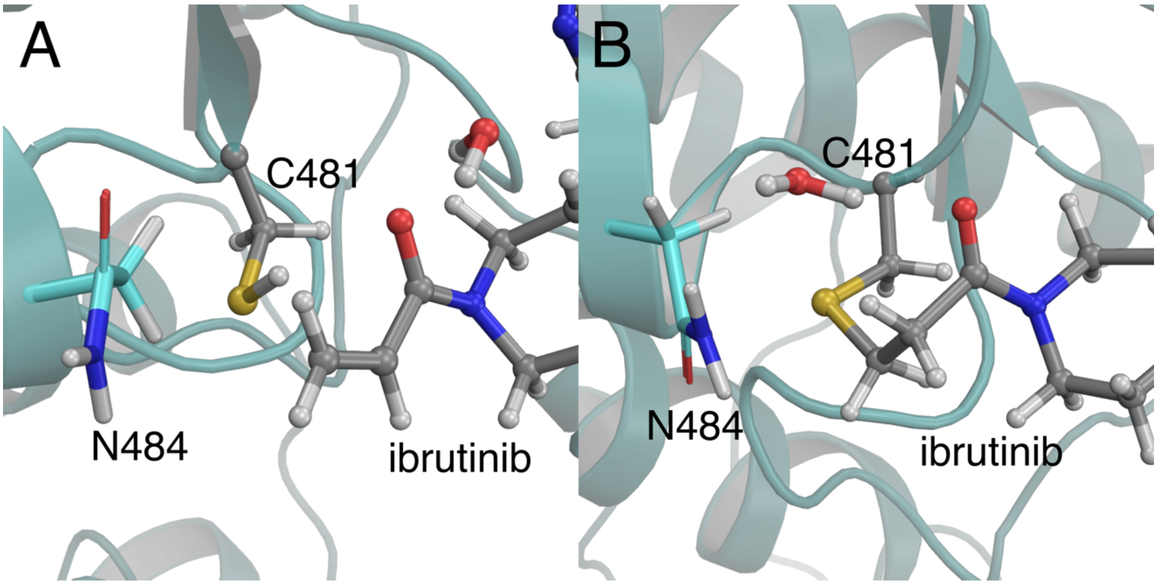

Figure S16. (A) Representative structure of ES from QM/MM umbrella sampling MD at the DFTB3/MM level. An additional water molecule is shown to highlight the solvent accessibility of the acrylamide warhead that can facilitate solvent-assisted tautomerisation. (B) Representative structure of EP from QM/MM umbrella sampling MD at the DFTB3/MM level. The additional water molecule shown was used to facilitate solvent-assisted proton transfer.

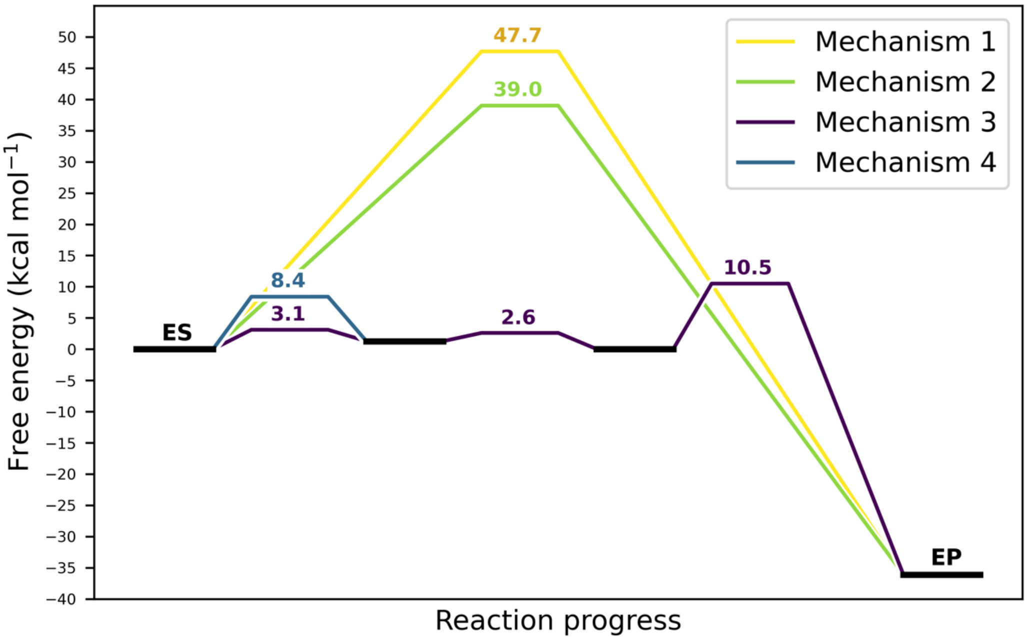

Figure S17. Free energy barrier heights of the four mechanistic pathways considered for the reaction between ibrutinib and C481 in BTK. Mechanism **3** is the lowest energy pathway with a rate-limiting step that corresponds to solvent-assisted keto-enol tautomerisation ($\Delta G^{\ddagger} = 10.5$ kcal mol^{-1}).

References

1 Gaussian 16, Revision A.01, M. J. Frisch, G. W. Trucks, H. B. Schlegel, G. E. Scuseria, M. A. Robb, J. R. Cheeseman, G. Scalmani, V. Barone, G. A. Petersson, H. Nakatsuji, M. C. X. Li, A. V. Marenich, J. Bloino, B. G. Janesko, R. Gomperts, B. Mennucci, H. P. Hratchian, J. V. Ortiz, A. F. Izmaylov, J. L. Sonnenberg, D. Williams-Young, F. Ding, F. Lipparini, F. Egidi, J. Goings, B. Peng, A. Petrone, T. Henderson, D. Ranasinghe, V. G. Zakrzewski, J. Gao, N. Rega, G. Zheng, W. Liang, M. Hada, M. Ehara, K. Toyota, R. Fukuda, J. Hasegawa, M. Ishida, T. Nakajima, Y. Honda, O. Kitao, H. Nakai, T. Vreven, K. Throssell, J. J. A. Montgomery, J. E. Peralta, F. Ogliaro, M. J. Bearpark, J. J. Heyd, E. N. Brothers, K. N. Kudin, V. N. Staroverov, T. A. Keith, R. Kobayashi, J. Normand, K. Raghavachari, A. P. Rendell, J. C. Burant, S. S. Iyengar, J. Tomasi, M. Cossi, J. M. Millam, M. Klene, C. Adamo, R. Cammi, J. W. Ochterski, R. L. Martin, K. Morokuma, O. Farkas, J. B. Foresman and D. J. Fox, Gaussian, Inc., Wallingford CT, 2016.

2 E. Awoonor-Williams, W. C. Isley, S. G. Dale, E. R. Johnson, H. Yu, A. D. Becke, B. Roux and C. N. Rowley, *J. Comput. Chem.*, 2020, **41**, 427–438.

3 J. M. Smith, Y. Jami Alahmadi and C. N. Rowley, *J. Chem. Theory Comput.*, 2013, **9**, 4860–4865.

4 A. T. Bender, A. Gardberg, A. Pereira, T. Johnson, Y. Wu, R. Grenningloh, J. Head, F. Morandi, P. Haselmayer and L. Liu-Bujalski, *Mol. Pharmacol.*, 2017, **91**, 208–219.

5 A. Šali and T. L. Blundell, *J. Mol. Biol.*, 1993, **234**, 779–815.

6 2018. Schrödinger Release 2018-3: Maestro, Schrödinger, LLC, New York, NY, .

7 C. R. Søndergaard, M. H. M. Olsson, M. Rostkowski and J. H. Jensen, *J. Chem. Theory Comput.*, 2011, **7**, 2284–2295.

8 M. H. M. Olsson, C. R. SØndergaard, M. Rostkowski and J. H. Jensen, *J. Chem. Theory Comput.*, 2011, **7**, 525–537.

9 E. Vanquelef, S. Simon, G. Marquant, E. Garcia, G. Klimerak, J. C. Delepine, P. Cieplak and F. Y. Dupradeau, *Nucleic Acids Res.*, 2011, **39**, W511–W517.

10 D.A. Case, K. Belfon, I.Y. Ben-Shalom, S.R. Brozell, D.S. Cerutti, I. T.E. Cheatham, V.W.D. Cruzeiro, T.A. Darden, R.E. Duke, G. Giambasu, M.K. Gilson, H. Gohlke, A.W. Goetz, R. Harris, S. Izadi, S.A. Izmailov, K. Kasavajhala, A. Kovalenko, R. Krasny, T. Kurtzman, T.S. Lee, S. LeGrand, J. L. P. Li, C. Lin, T. Luchko, R. Luo, V. Man, K.M. Merz, Y. Miao, O. Mikhailovskii, G. Monard, H. Nguyen, A. Onufriev, S. P. F.Pan, R. Qi, D.R. Roe, A. Roitberg, C. Sagui, S. Schott-Verdugo, J. Shen, C.L. Simmerling, N.R.Skrynnikov, J. Smith, J. Swails, R.C. Walker, J. Wang, L. Wilson, R.M. Wolf, X. Wu, Y. Xiong, Y. Xue, D.M. York and P.A. Kollman, (2018), AMBER 2018, University of California, San Francisco.

11 Solvate | Max Planck Institute for Biophysical Chemistry, https://www.mpibpc.mpg.de/grubmueller/solvate, (accessed 30 October 2020).

12 K. Świderek and V. Moliner, *Chem. Sci.*, 2020, **11**, 10626–10630.

13 A. Grossfield, WHAM: the weighted histogram analysis method, http://membrane.urmc.rochester.edu/content/wham/, (accessed 15 July 2020).

14 S. Kumar, J. M. Rosenberg, D. Bouzida, R. H. Swendsen and P. A. Kollman, *J. Comput. Chem.*, 1992, **13**, 1011–1021.

15 J. Kästner and W. Thiel, *J. Chem. Phys.*, 2006, **124**, 234106.

16 J. Kästner, *Wiley Interdiscip. Rev. Comput. Mol. Sci.*, 2011, **1**, 932–942.
